# (6,6′-Dimethyl-2,2′-bipyridine-κ^2^ *N*,*N*′)diiodidomercury(II)

**DOI:** 10.1107/S1600536811004041

**Published:** 2011-02-05

**Authors:** Robabeh Alizadeh, Sara Seifi, Vahid Amani

**Affiliations:** a School of Chemistry, Damghan University, Damghan, Iran; b Islamic Azad University, Shahr-e-Rey Branch, Tehran, Iran

## Abstract

In the title complex, [HgI_2_(C_12_H_12_N_2_)], the Hg^II^ atom has a distorted tetra­hedral coordination formed by two N atoms of the 6,6′-dimethyl-2,2′-bipyridine ligand and two terminal I atoms [N—Hg—N = 70.1 (2) and I—Hg—I = 130.59 (3)°]. The crystal packing features π–π contacts between the pyridine rings of adjacent mol­ecules [centroid–centroid distance = 3.773 (5) Å] and also between a pyridine ring of one mol­ecule and the five-membered chelate ring of an adjacent mol­ecule [centroid–centroid distance = 3.668 (4) Å].

## Related literature

For the structures of metal complexes with a 6,6′-dimethyl-2,2′-bipyridine ligand, see: Akbarzadeh Torbati *et al.* (2010 ▶); Alizadeh *et al.* (2010 ▶); Alizadeh, Kalateh, Ebadi *et al.* (2009 ▶); Alizadeh, Kalateh, Khoshtarkib *et al.* (2009 ▶); Alizadeh, Khoshtarkib *et al.* (2009 ▶); Itoh *et al.* (2005 ▶); Kou *et al.* (2008 ▶); Onggo *et al.* (2005 ▶).
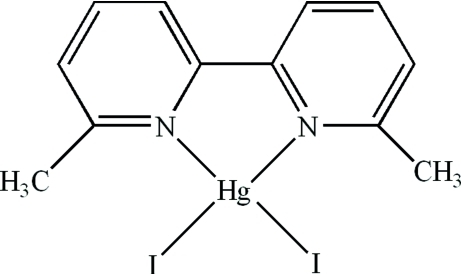


## Experimental

### 

#### Crystal data

[HgI_2_(C_12_H_12_N_2_)]
*M* *_r_* = 638.63
Monoclinic, 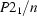

*a* = 8.8096 (18) Å
*b* = 12.025 (2) Å
*c* = 14.693 (3) Å
β = 101.88 (3)°
*V* = 1523.2 (5) Å^3^
*Z* = 4
Mo *K*α radiation
μ = 14.14 mm^−1^
*T* = 298 K
0.16 × 0.15 × 0.12 mm

#### Data collection

Bruker APEXII CCD area-detector diffractometer
Absorption correction: multi-scan (*SADABS*; Bruker, 2001 ▶) *T*_min_ = 0.128, *T*_max_ = 0.186
9361 measured reflections
4057 independent reflections
3409 reflections with *I* > 2σ(*I*)
*R*_int_ = 0.051

#### Refinement

*R*[*F* ^2^ > 2σ(*F* ^2^)] = 0.040
*wR*(*F* ^2^) = 0.095
*S* = 1.10
4057 reflections
154 parameters
H-atom parameters constrained
Δρ_max_ = 1.22 e Å^−3^
Δρ_min_ = −1.23 e Å^−3^

### 

Data collection: *APEX2* (Bruker, 2007 ▶); cell refinement: *SAINT* (Bruker, 2007 ▶); data reduction: *SAINT*; program(s) used to solve structure: *SHELXTL* (Sheldrick, 2008 ▶); program(s) used to refine structure: *SHELXTL*; molecular graphics: *ORTEP-3 for Windows* (Farrugia, 1997 ▶); software used to prepare material for publication: *WinGX* (Farrugia, 1999 ▶).

## Supplementary Material

Crystal structure: contains datablocks I, global. DOI: 10.1107/S1600536811004041/ya2136sup1.cif

Structure factors: contains datablocks I. DOI: 10.1107/S1600536811004041/ya2136Isup2.hkl

Additional supplementary materials:  crystallographic information; 3D view; checkCIF report

## Figures and Tables

**Table 1:** Selected bond lengths (Å)

N1—Hg1	2.380 (5)
N2—Hg1	2.381 (6)
I1—Hg1	2.6503 (10)
I2—Hg1	2.6876 (7)

**Fig. 1.**
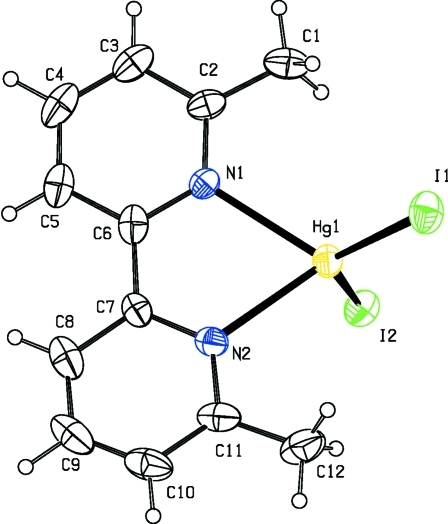
Molecular structure of the title compound;. displacement ellipsoids are drawn at the 30% probability level.

**Fig. 2.**
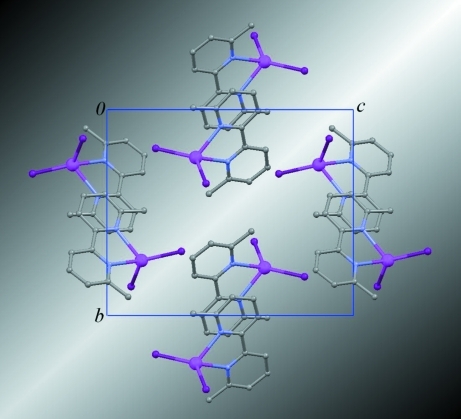
Packing diagram for the crystal of the title compound viewed along the a axis.

## supplementary crystallographic information

### Comment

6,6'-Dimethyl-2,2'-bipyridine (6,6'-dmbipy) is a rather widely used bidentate ligand, and complexes of different metals with 6,6'-dmbipy have been prepared, *e.g.* those of cobalt (Akbarzadeh Torbati *et al.*, 2010),cadmium (Alizadeh *et al.*, 2010),zinc (Alizadeh, Kalateh, Ebadi *et al.*, 2009; Alizadeh, Kalateh, Khoshtarkib *et al.*, 2009; Alizadeh, Khoshtarkib *et al.*, 2009), copper (Itoh *et al.*, 2005), nickel (Kou *et al.*, 2008), and ruthenium (Onggo *et al.*, 2005). We report herein the synthesis and first crystal structure of the mercury complex of this ligand (Fig. 1).

The Hg1 atom has a distorted tetrahedral coordination formed by atoms N1 and N2 of the 6,6'-dimethyl-2,2'-bipyridine ligand and terminal I1 and I2 atoms [N—Hg—N 70.1 (2)°; I—Hg—I 130.59 (3)°; see Table 1 for bond lengths involving Hg1].

In the crystal structure, intermolecular /*p*-/p contacts (Fig. 2) between the pyridine rings and also between pyridine ring and chelate ring of the adjacent molecules may stabilize the structure: the centroid-centroid distances *Cg*1—*Cg*2^i^ and *Cg*2—*Cg*3^i^ are equal to 3.668 (4) and 3.773 (5)Å respectively [*Cg*1, *Cg*2 and *Cg*3 represent centroids of the rings (Hg1/N1/C6/C7/N2), (N1/C2—C6), and (N2/C7—C11); symmetry code (i): 2 - *x*,1 - *y*,-*z*].

### Experimental

For the preparation of the title compound, a solution of 6,6'-dimethyl-2,2'-bipyridine (0.28 g, 1.50 mmol) in acetonitrile (10 ml) was added to a solution of HgI_2_ (0.68 g, 1.50 mmol) in methanol (10 ml), and the resulting colorless mixture was stirred for 30 min at 313 K. It was then left to evaporate slowly at room temperature. After six days, colorless prismatic crystals of the title compound, suitable for X-ray diffraction experiment, were isolated (yield 0.71 g; 74.1%).

### Refinement

All H atoms were positioned geometrically, with C—H 0.93 and 0.96 Å for aromatics and methyl hydrogen atoms, respectively, and constrained to ride on their parent atoms, with *U*_iso_(H)=1.2*U*_eq_. The highest residual density peak and the deepest hole (1.22 and -1.23 e A°^-3^) are located at distances of 0.83 and 0.82 Å from the Hg1 atom respectively.

### Crystal data

**Table:**

[HgI_2_(C_12_H_12_N_2_)]	*F*(000) = 1136
*M**_r_* = 638.63	*D*_x_ = 2.785 Mg m^−^^3^
Monoclinic, *P*2_1_/*n*	Mo *K*α radiation, λ = 0.71073 Å
Hall symbol: -P 2yn	Cell parameters from 1223 reflections
*a* = 8.8096 (18) Å	θ = 2.2–29.2°
*b* = 12.025 (2) Å	µ = 14.14 mm^−^^1^
*c* = 14.693 (3) Å	*T* = 298 K
β = 101.88 (3)°	Prism, colorless
*V* = 1523.2 (5) Å^3^	0.16 × 0.15 × 0.12 mm
*Z* = 4	

### Data collection

**Table:**

Bruker APEXII CCD area-detector diffractometer	4057 independent reflections
Radiation source: fine-focus sealed tube	3409 reflections with *I* > 2σ(*I*)
graphite	*R*_int_ = 0.051
φ and ω scans	θ_max_ = 29.2°, θ_min_ = 2.2°
Absorption correction: multi-scan (*SADABS*; Bruker, 2001)	*h* = −11→12
*T*_min_ = 0.128, *T*_max_ = 0.186	*k* = −16→13
9361 measured reflections	*l* = −20→17

### Refinement

**Table:**

Refinement on *F*^2^	Primary atom site location: structure-invariant direct methods
Least-squares matrix: full	Secondary atom site location: difference Fourier map
*R*[*F*^2^ > 2σ(*F*^2^)] = 0.040	Hydrogen site location: inferred from neighbouring sites
*wR*(*F*^2^) = 0.095	H-atom parameters constrained
*S* = 1.10	*w* = 1/[σ^2^(*F*_o_^2^) + (0.0397*P*)^2^ + 3.5854*P*] where *P* = (*F*_o_^2^ + 2*F*_c_^2^)/3
4057 reflections	(Δ/σ)_max_ = 0.001
154 parameters	Δρ_max_ = 1.22 e Å^−^^3^
0 restraints	Δρ_min_ = −1.23 e Å^−^^3^

### Special details

**Table:**

Geometry. All e.s.d.'s (except the e.s.d. in the dihedral angle between two l.s. planes) are estimated using the full covariance matrix. The cell e.s.d.'s are taken into account individually in the estimation of e.s.d.'s in distances, angles and torsion angles; correlations between e.s.d.'s in cell parameters are only used when they are defined by crystal symmetry. An approximate (isotropic) treatment of cell e.s.d.'s is used for estimating e.s.d.'s involving l.s. planes.
Refinement. Refinement of *F*^2^ against ALL reflections. The weighted *R*-factor *wR* and goodness of fit *S* are based on *F*^2^, conventional *R*-factors *R* are based on *F*, with *F* set to zero for negative *F*^2^. The threshold expression of *F*^2^ > σ(*F*^2^) is used only for calculating *R*-factors(gt) *etc*. and is not relevant to the choice of reflections for refinement. *R*-factors based on *F*^2^ are statistically about twice as large as those based on *F*, and *R*- factors based on ALL data will be even larger.

### Fractional atomic coordinates and isotropic or equivalent isotropic displacement parameters (Å^2^)

**Table:**

	*x*	*y*	*z*	*U*_iso_*/*U*_eq_	
C1	0.6805 (9)	0.4904 (8)	0.1498 (7)	0.065 (2)	
H1A	0.6238	0.5560	0.1595	0.077*	
H1B	0.7749	0.4868	0.1960	0.077*	
H1C	0.6186	0.4258	0.1547	0.077*	
C2	0.7174 (7)	0.4943 (6)	0.0569 (5)	0.0493 (16)	
C3	0.6678 (9)	0.4111 (7)	−0.0091 (6)	0.061 (2)	
H3	0.6079	0.3520	0.0042	0.073*	
C4	0.7097 (10)	0.4188 (7)	−0.0941 (7)	0.069 (2)	
H4	0.6773	0.3644	−0.1388	0.083*	
C5	0.7983 (9)	0.5055 (7)	−0.1137 (6)	0.060 (2)	
H5	0.8272	0.5100	−0.1710	0.072*	
C6	0.8444 (7)	0.5869 (6)	−0.0461 (5)	0.0478 (15)	
C7	0.9441 (7)	0.6807 (6)	−0.0608 (4)	0.0445 (14)	
C8	0.9964 (10)	0.6955 (9)	−0.1438 (6)	0.066 (2)	
H8	0.9628	0.6477	−0.1936	0.079*	
C9	1.0961 (13)	0.7794 (10)	−0.1518 (8)	0.080 (3)	
H9	1.1294	0.7900	−0.2073	0.096*	
C10	1.1474 (10)	0.8489 (8)	−0.0772 (8)	0.074 (3)	
H10	1.2190	0.9046	−0.0812	0.089*	
C11	1.0915 (9)	0.8353 (7)	0.0045 (7)	0.0592 (19)	
C12	1.1391 (11)	0.9092 (8)	0.0862 (8)	0.079 (3)	
H12A	1.1882	0.8659	0.1389	0.094*	
H12B	1.0493	0.9456	0.0998	0.094*	
H12C	1.2106	0.9640	0.0728	0.094*	
N1	0.8032 (6)	0.5792 (4)	0.0366 (4)	0.0399 (11)	
N2	0.9903 (6)	0.7526 (5)	0.0104 (4)	0.0452 (12)	
I1	1.05284 (6)	0.68838 (5)	0.30203 (4)	0.06506 (16)	
I2	0.61128 (6)	0.85868 (4)	0.09788 (4)	0.05659 (14)	
Hg1	0.86805 (3)	0.73253 (2)	0.139654 (19)	0.04718 (9)	

### Atomic displacement parameters (Å^2^)

**Table:**

	*U*^11^	*U*^22^	*U*^33^	*U*^12^	*U*^13^	*U*^23^
C1	0.051 (4)	0.060 (5)	0.081 (6)	−0.010 (3)	0.009 (4)	0.019 (4)
C2	0.041 (3)	0.037 (3)	0.066 (4)	0.003 (2)	0.003 (3)	0.005 (3)
C3	0.047 (3)	0.045 (4)	0.082 (6)	0.004 (3)	−0.005 (4)	−0.010 (4)
C4	0.062 (4)	0.056 (5)	0.077 (6)	0.008 (4)	−0.012 (4)	−0.027 (4)
C5	0.057 (4)	0.068 (5)	0.051 (4)	0.014 (4)	0.004 (3)	−0.018 (4)
C6	0.041 (3)	0.058 (4)	0.042 (3)	0.019 (3)	0.002 (2)	−0.005 (3)
C7	0.045 (3)	0.051 (4)	0.039 (3)	0.014 (3)	0.012 (2)	0.006 (3)
C8	0.066 (5)	0.088 (6)	0.047 (4)	0.020 (4)	0.020 (4)	0.009 (4)
C9	0.087 (6)	0.094 (8)	0.070 (6)	0.019 (6)	0.041 (5)	0.034 (6)
C10	0.062 (5)	0.065 (6)	0.107 (8)	0.009 (4)	0.046 (5)	0.028 (5)
C11	0.050 (4)	0.045 (4)	0.088 (6)	0.003 (3)	0.026 (4)	0.017 (4)
C12	0.074 (6)	0.053 (5)	0.116 (8)	−0.016 (4)	0.034 (6)	−0.012 (5)
N1	0.038 (2)	0.036 (3)	0.045 (3)	0.005 (2)	0.006 (2)	−0.002 (2)
N2	0.045 (3)	0.040 (3)	0.054 (3)	0.006 (2)	0.021 (2)	0.006 (2)
I1	0.0656 (3)	0.0743 (4)	0.0493 (3)	0.0076 (3)	−0.0020 (2)	−0.0064 (2)
I2	0.0534 (2)	0.0536 (3)	0.0609 (3)	0.0024 (2)	0.0073 (2)	−0.0054 (2)
Hg1	0.05023 (14)	0.04953 (15)	0.04286 (13)	−0.00229 (11)	0.01209 (10)	−0.00399 (11)

### Geometric parameters (Å, °)

**Table:**

C1—C2	1.468 (12)	C8—C9	1.359 (15)
C1—H1A	0.9600	C8—H8	0.9300
C1—H1B	0.9600	C9—C10	1.379 (16)
C1—H1C	0.9600	C9—H9	0.9300
C2—N1	1.340 (9)	C10—C11	1.398 (13)
C2—C3	1.399 (10)	C10—H10	0.9300
C3—C4	1.377 (14)	C11—N2	1.351 (9)
C3—H3	0.9300	C11—C12	1.483 (14)
C4—C5	1.367 (13)	C12—H12A	0.9600
C4—H4	0.9300	C12—H12B	0.9600
C5—C6	1.394 (10)	C12—H12C	0.9600
C5—H5	0.9300	N1—Hg1	2.380 (5)
C6—N1	1.341 (9)	N2—Hg1	2.381 (6)
C6—C7	1.473 (11)	I1—Hg1	2.6503 (10)
C7—N2	1.354 (9)	I2—Hg1	2.6876 (7)
C7—C8	1.401 (10)		
			
C2—C1—H1A	109.5	C8—C9—C10	119.5 (9)
C2—C1—H1B	109.5	C8—C9—H9	120.2
H1A—C1—H1B	109.5	C10—C9—H9	120.2
C2—C1—H1C	109.5	C9—C10—C11	119.8 (9)
H1A—C1—H1C	109.5	C9—C10—H10	120.1
H1B—C1—H1C	109.5	C11—C10—H10	120.1
N1—C2—C3	120.0 (8)	N2—C11—C10	119.7 (9)
N1—C2—C1	118.4 (7)	N2—C11—C12	118.1 (8)
C3—C2—C1	121.6 (7)	C10—C11—C12	122.3 (8)
C4—C3—C2	118.5 (8)	C11—C12—H12A	109.5
C4—C3—H3	120.8	C11—C12—H12B	109.5
C2—C3—H3	120.8	H12A—C12—H12B	109.5
C5—C4—C3	120.9 (8)	C11—C12—H12C	109.5
C5—C4—H4	119.5	H12A—C12—H12C	109.5
C3—C4—H4	119.5	H12B—C12—H12C	109.5
C4—C5—C6	118.7 (8)	C2—N1—C6	121.6 (6)
C4—C5—H5	120.6	C2—N1—Hg1	121.4 (5)
C6—C5—H5	120.6	C6—N1—Hg1	116.7 (5)
N1—C6—C5	120.2 (8)	C11—N2—C7	121.2 (7)
N1—C6—C7	117.8 (6)	C11—N2—Hg1	122.6 (6)
C5—C6—C7	121.9 (7)	C7—N2—Hg1	116.0 (4)
N2—C7—C8	119.4 (7)	N1—Hg1—N2	70.1 (2)
N2—C7—C6	118.0 (6)	N1—Hg1—I1	116.12 (12)
C8—C7—C6	122.6 (7)	N2—Hg1—I1	116.33 (14)
C9—C8—C7	120.3 (9)	N1—Hg1—I2	102.20 (12)
C9—C8—H8	119.8	N2—Hg1—I2	104.98 (13)
C7—C8—H8	119.8	I1—Hg1—I2	130.59 (3)
			
N1—C2—C3—C4	0.3 (10)	C5—C6—N1—Hg1	−174.1 (5)
C1—C2—C3—C4	−178.5 (7)	C7—C6—N1—Hg1	8.4 (7)
C2—C3—C4—C5	0.2 (12)	C10—C11—N2—C7	1.4 (11)
C3—C4—C5—C6	−0.7 (12)	C12—C11—N2—C7	−178.7 (7)
C4—C5—C6—N1	0.6 (10)	C10—C11—N2—Hg1	−173.7 (6)
C4—C5—C6—C7	177.9 (6)	C12—C11—N2—Hg1	6.2 (10)
N1—C6—C7—N2	1.0 (8)	C8—C7—N2—C11	−3.1 (10)
C5—C6—C7—N2	−176.4 (6)	C6—C7—N2—C11	174.8 (6)
N1—C6—C7—C8	178.7 (6)	C8—C7—N2—Hg1	172.3 (5)
C5—C6—C7—C8	1.3 (10)	C6—C7—N2—Hg1	−9.8 (7)
N2—C7—C8—C9	1.8 (11)	C2—N1—Hg1—N2	176.3 (5)
C6—C7—C8—C9	−175.9 (8)	C6—N1—Hg1—N2	−9.7 (4)
C7—C8—C9—C10	1.1 (14)	C2—N1—Hg1—I1	65.9 (5)
C8—C9—C10—C11	−2.7 (15)	C6—N1—Hg1—I1	−120.0 (4)
C9—C10—C11—N2	1.5 (13)	C2—N1—Hg1—I2	−82.0 (4)
C9—C10—C11—C12	−178.4 (9)	C6—N1—Hg1—I2	92.1 (4)
C3—C2—N1—C6	−0.4 (9)	C11—N2—Hg1—N1	−174.6 (6)
C1—C2—N1—C6	178.5 (6)	C7—N2—Hg1—N1	10.1 (4)
C3—C2—N1—Hg1	173.4 (5)	C11—N2—Hg1—I1	−64.5 (6)
C1—C2—N1—Hg1	−7.8 (8)	C7—N2—Hg1—I1	120.2 (4)
C5—C6—N1—C2	−0.1 (9)	C11—N2—Hg1—I2	87.6 (5)
C7—C6—N1—C2	−177.5 (5)	C7—N2—Hg1—I2	−87.7 (4)
